# Dual-wavelength multiplexed metasurface holography based on two-photon polymerization lithography

**DOI:** 10.1515/nanoph-2024-0705

**Published:** 2025-03-10

**Authors:** Lei Zhang, Hongbo Wang, Qiang Jiang, Liangzhi Han, Xuedian Zhang, Songlin Zhuang

**Affiliations:** School of Optical-Electrical and Computer Engineering, University of Shanghai for Science and Technology, Shanghai 200093, China; Beijing Engineering Research Center of Mixed Reality and Advanced Display, School of Optics and Photonics, 47833 Beijing Institute of Technology, Beijing 100081, China; Nanoscribe China Co., Ltd., Shanghai 200233, China

**Keywords:** metasurface, hologram, two-photon polymerization, nanophotonics

## Abstract

Two-photon polymerization (TPP) lithography can process 3D micro–nano structures with high precision and has wide applications in the fields of micro-optics. Metasurfaces can flexibly control electromagnetic fields at subwavelength scale, achieving functions such as multidimensional multiplexing holography and achromatic imaging. Meta-devices are usually fabricated via EBL-based process, which is complex and difficult to fabricate meta-devices composed of meta-atoms with different heights. Here, we design a color dual-wavelength metasurface hologram without spatial multiplexing. By combining the propagation phase and the geometric phase, the phase response of two wavelengths is achieved in the same polarization state, and the metasurface is prepared using TPP 3D laser printing technology. The experimentally reconstructed images are consistent with theoretical predictions. This not only verifies the feasibility of this 3D printing technology in the preparation of metasurface samples operating in visible band but also provides potential applications in holographic display, optical encryption, anticounterfeiting, and other fields.

## 1 Introduction

Metasurface is considered a technology that can bring about a disruptive revolution in optical components and has attracted widespread attention in recent years. As an ultrathin planar optical device consisting of an array of subwavelength antennas, metasurface can flexibly control physical properties such as amplitude, phase, and polarization of electromagnetic waves by extremely thin structures [1], [2], [3], [4], [5], [6]. This unique light field control advantage allows it to be used in various elements such as metalenses [7], [8], polarizers [9], vortex beam generators [10], holographic devices [11], [12], etc. It is even expected to replace most of the existing bulky and single-function optical devices.

The advantages of multidimensional control of metasurfaces at the subwavelength scale can be more obvious in holographic devices, which aiming to recording information as much as possible with multiplexing technique. In computer-generated holographic display, the pixel size of the spatial light modulator (SLM) is almost an order of magnitude larger than that of visible light, leading to higher-order diffraction in the reconstructed images and a small viewing angle. In addition, to realize high-resolution color display, SLMs often require space-multiplexing or time-sequence strategy, thus sacrifice the spatial resolution or refresh rate. Metasurface, a new kind of SLM that flexibly control electromagnetic waves at a subwavelength scale across multiple dimensions, can provide greater freedom in designing high-capacity holograms that modulate the amplitude, phase, and polarization at different wavelengths simultaneously, along with an expanded viewing angle and suppressed higher-order diffractions [13], [14]. Recently, full-color metasurface-based holographic display using surface plasmons and dielectric materials have been proposed, demonstrating significant potential for enhancing information display and storage capacity. Among them, those that use spatial multiplexing strategies will sacrifice spatial resolution [15], polarization multiplexing need to control the polarization state to select colors [16], [17], [18], and those that use different diffraction order positions to overlap will sacrifice energy [19]. To take full advantages of the multidimensional control of light offered by metasurfaces, researchers have moved toward multiwavelength metasurface hologram based on single meta-atom [20], [21].

From a processing perspective, metasurface devices are typically fabricated by electron beam lithography (EBL) combined with etching processes. However, this technology involves complex workflows that may accumulate significant errors, and processing complex meta-atoms of different heights is also a huge challenge. With the development of high-precision additive manufacturing technology, two-photon polymerization (TPP) printing technology has been studied in the fabrication of metasurface devices in recent years. For example, variable height metasurface, multilayered structures, metasurface of the helical plane [22], [23], [24], etc. Different from EBL, TPP technology has great advantages in processing metasurfaces of arbitrary shapes by solidifying the photoresist material point by point at the subwavelength scale, which can give full play to the multidimensional light field control advantages of metasurfaces. Furthermore, this method only requires exposure and development steps, significantly simplifying the manufacturing process compared to EBL-based processes.

**Figure 1:**
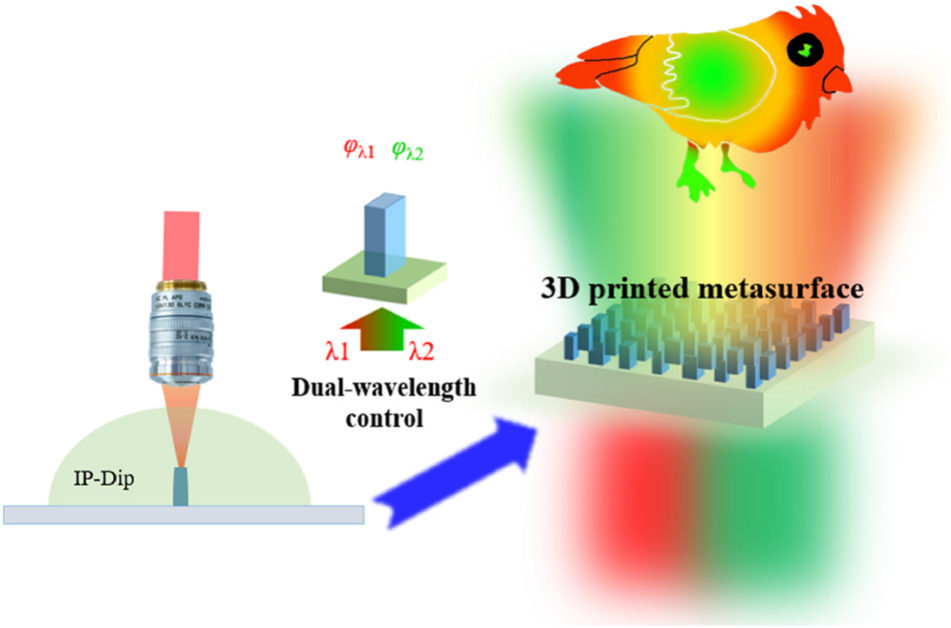
Schematic illustration of 3D-printed metasurface color holographic display.

In this work, we first design a color metasurface hologram using single-atom controlled dual-wavelength phase modulation nanostructures based on the dispersion properties of propagation phase and geometric phase, as is shown in Figure 1. By introducing rotation angle, *m* ^2^ phase pairs corresponding to two wavelengths can be decreased to *m* phase pairs, and the high-resolution features can be preserved without the need for spatial and polarization multiplexing. The designed fine structures are then fabricated via a commercial TPP 3D printing system. The experimental reconstructions of metasurface samples are in good agreement with our expectations. On the one hand, our work demonstrates the feasibility of 3D printing metasurface holographic samples that work in the visible light region; on the other hand, the designed metasurfaces have potential applications in areas such as optical encryption, color holography, and other metasurface devices requiring multiwavelength phase and amplitude modulation.

## 2 Analysis and simulation

### 2.1 Metasurface design

According to the PB-phase theory, when circularly polarized light $\left(\begin{array}{c} 1\\ \sigma \text{i} \end{array}\right)$ passes through an anisotropic nanorod, the complex amplitude of the output light can be expressed as

(1) $$E_{\text{out}}=\frac{T_{L}+T_{s}}{2}\left(\begin{array}{c} 1\\ \sigma i \end{array}\right)+\frac{T_{L}-T_{s}}{2}e^{i2\sigma \alpha }\left(\begin{array}{c} 1\\ -\sigma i \end{array}\right)$$

Where *α* is the rotation angle of the nanopillar, *σ* denotes the rotational orientation of the circularly polarized light (*σ* = 1, left-handed circularly polarized light (LCP); *σ* = −1, right-handed circularly polarized light (RCP)), *T*_*L/S*_ is the complex transmission coefficients (*T*_*L/S*_ = *A*_*L/S*_exp (−i*φ*_*L/S*_), the amplitude *A*_*L/S*_ and phase *φ*_*L/S*_ are determined by the geometric size parameters of meta-atom) along the long/short axis of the nanorod, respectively. In equation (1), the second term describes the circular polarization in the outgoing light that is opposite to the polarization state of the incident light, which carries a phase change of *φ*_PB_ = 2*σα*. The amplitude of this term is determined by *T*_*L*_ and *T*_*S*_, while the phase is governed by both *φ*_PB_ and dynamic phase *φ*_*LS*_. Due to the dispersion of meta-atom, *φ*_*LS*_ is sensitive to wavelength, while *φ*_PB_ is insensitive to wavelength. Thus, *φ*_PB_ can be used as a bias for realizing arbitrary phase pair of the meta-atom operating at two wavelengths. Assuming that the phase response of one nanopillar is *φ*_1_ (*λ*_1_) for one wavelength and *φ*_2_ (*λ*_2_) for another wavelength, a phase pair [*φ*_1_ (*λ*_1_), *φ*_2_ (*λ*_2_)] is formed, and when the nanopillar is rotated by an angle of *α*, the phase pair can be written as [*φ*_1_(*λ*_1_) +2*α*, *φ*_2_(*λ*_2_) +2*α*].

**Figure 2:**
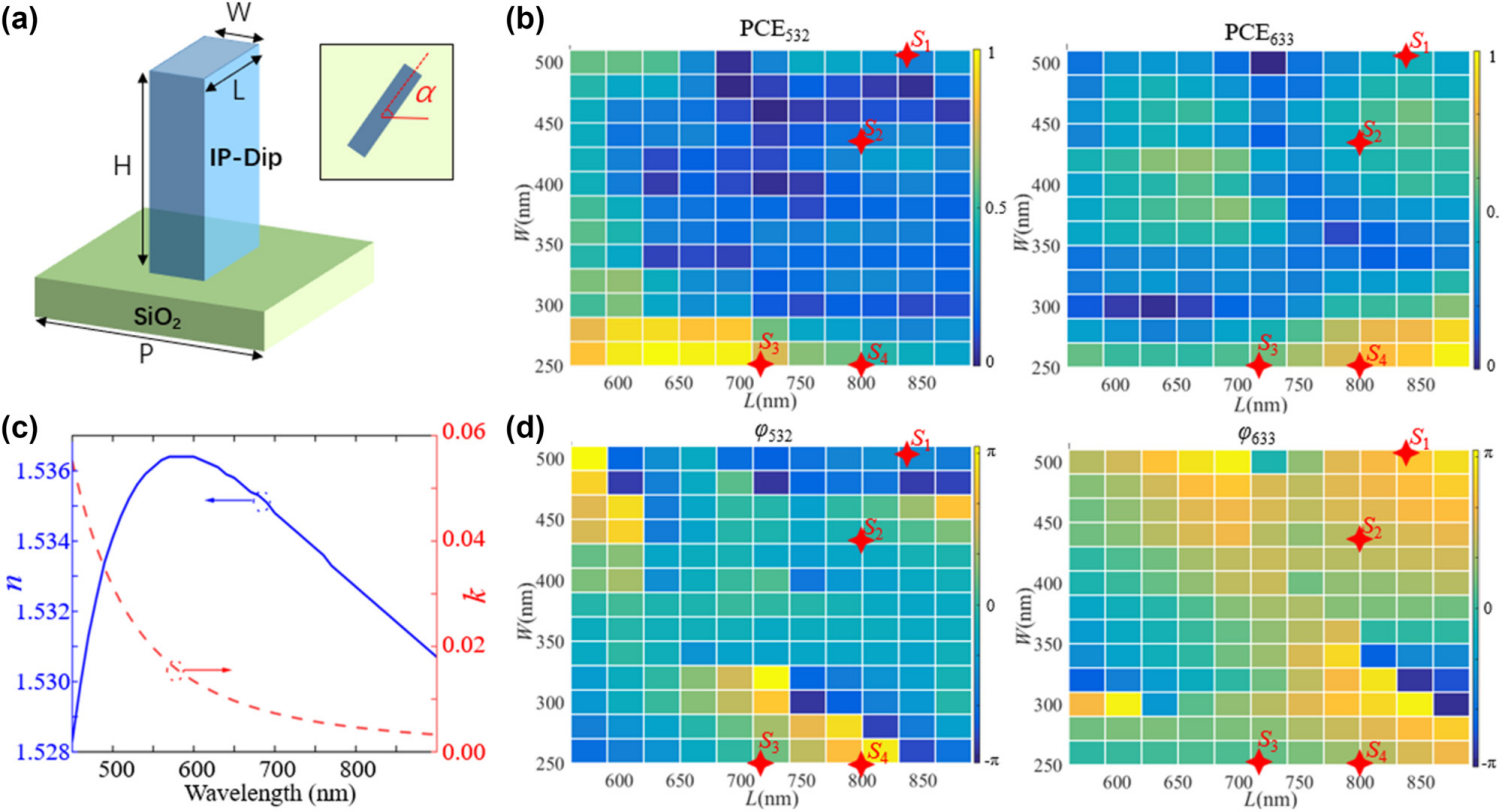
Meta-atom and its PCE/phase response. (a) Schematic diagram of meta-atom.(c) Refractive index of the IP-Dip material. (b) and (d) PCE and dynamic phase of the cross circularly polarized light at *λ* = 532 nm and *λ* = 633 nm, respectively. The length L varies from 600 nm to 890 nm, and the width W varies from 250 nm to 510 nm.Four red stars represent meta-atoms S1, S2, S3, S4 used in this work.

The metasurface used in this work consists of cured photoresist nanobricks on the surface of SiO_2_ substrate, as shown in Figure 2(a). The refractive index of the cured IP-Dip polymer material at the wavelength range of 450 nm–900 nm is shown in Figure 2(c). Since the refractive index is not that high (∼1.5), to maintain a relatively high polarization conversion efficiency (PCE), we optimized the parameters of the meta-atom, and set the period P to 1.25 µm and the height H to 4.2 µm, after referring to the parameters of other works using the same photoresist [25]. By parameter sweeping *L* and *W* via Lumerical FDTD software, the PCE and dynamic phase at two operating wavelengths of 532 nm and 633 nm can be obtained, as shown in Figure 2(b) and (d). The dynamic phase of the meta-atom at two wavelengths covers the range of [−π, π], and the PCE changes from 0 to 1, which provides possible structures for encoding holograms corresponding to the two wavelengths.

**Figure 3:**
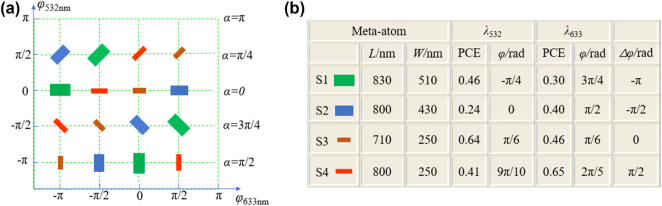
The four selected structures achieve phase control at two wavelengths. (a) Sixteen phase pairs corresponding to two wavelengths can be encoded by rotating the selected meta-atoms. (b) The geometric parameters of selected meta-atoms S1, S2, S3, S4 and their PCE and phase corresponding to two wavelengths.

For dual-wavelength holography, assuming that the two sets of holograms corresponding to two wavelengths are composed of *m*-level phase, then *m* ^2^ meta-atoms are required to be found to encode all phase pairs. Considering the periodicity of the phase and the insensitivity of the PB phase to wavelength, in fact, we can reduce the number of required meta-atoms to *m* by rotating meta-atoms that conform to a specific phase distribution. Specifically, if *m* is set to 4, then four structures in which the propagation phase difference at two different wavelengths spaced linearly by π/2 are required. By carefully searching in the amplitude and phase response library in Figure 2, meta-atoms S1, S2, S3, S4 that meet the requirements are marked with red asterisks in Figure 2(b) and (d). We try our best to find meta-atoms with suitable phase distribution and high PCE, and their corresponding geometric parameters and complex amplitude responses are shown in Figure 3(b). For instance, the nanopillar S1, when not rotated, yields a propagation phase of −π/4 at *λ* = 532 nm and 3π/4 at *λ* = 633 nm. The propagation phase pair can be expressed as (−π/4,3π/4). When introducing a rotation angle of π/4, the phase pair becomes (π/4,5π/4) as each phase value increases by π/2. Likewise, other phase pairs such as (3π/4,7π/4), (5π/4,9π/4) can also be generated. By using PB phase to the selected meta-atoms, all 16 phase pairs can be realized, as shown in Figure 3(a). Thus, the meta-devices constructed from the selected meta-atoms can achieve light field control functions at dual wavelengths.

### 2.2 Simulation

**Figure 4:**
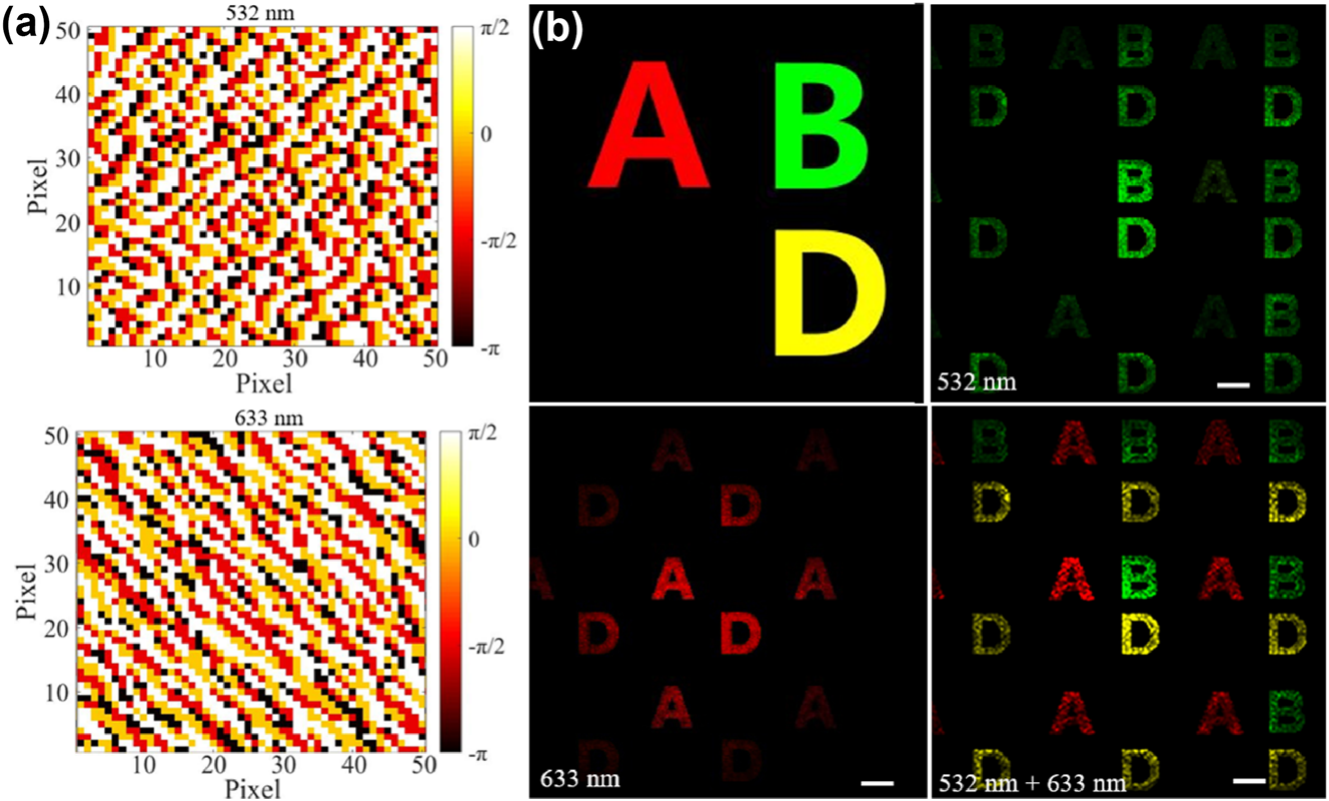
Hologram phase distribution and simulation reconstruction result of color images at two wavelengths. (a) Phase distribution of the two holograms corresponding to 532 nm and 633 nm. (b) Target image and simulated reconstructed images at different wavelengths. Scale bars: 35 μm.

A metasurface hologram for colorful display is designed to verify the dual-wavelength phase modulation capability of the proposed meta-atoms. The classic Gerchberg–Saxton (GS) algorithm is applied for calculating the holograms of these two different wavelengths, and the phase maps are quantized into four levels, as shown in Figure 4(a) [26]. Utilizing the four types of selected meta-atoms, two sets of phase maps can be encoded by the metasurface. To verify the reconstruction results in simulate via Lumerical software, a 50 × 50 pixels metasurface is used considering memory limitations of computer. In the simulation, capital letters ABD (Figure 4(b)) of different colors are used as target images, the corresponding reconstructed images from metasurface are shown in Figure 4. Under the irradiation of light waves with a wavelength of 532 nm, the simulation results show reconstructed images of multiple diffraction orders, which is caused by the metasurface period being larger than the operating wavelength. As shown in Figure 4(b), the first-order diffraction image has the greatest intensity compared with other orders. Under illumination with a wavelength of 532 nm, the expected green letters “B” and “D” were successfully reconstructed. Although the undesired letter “A” appears, the intensity is relatively small. While under the illumination of light at 633 nm, the reconstructed image precisely displayed the desired letters red “A” and “D,” without any evidence of crosstalk. The crosstalk in these two images may be caused by suboptimal polarization conversion efficiency within the structure. In addition, when calculating the phase response of meta-atoms, periodic boundary conditions are used, but the rotation and size differences between adjacent structures in the actual metasurface will affect the actual phase changes. This disrupts the phase distribution of the design, which can also cause crosstalk issues. If one wants to further mitigate this disruption, strategies such as inverse design can be employed to compute and optimize the electromagnetic response across the entire metasurface [27]. When two wavelengths are incident simultaneously, the color distribution of the reconstructed image ABD is highly consistent with the target image.

## 3 Experiment and discussion

**Figure 5:**
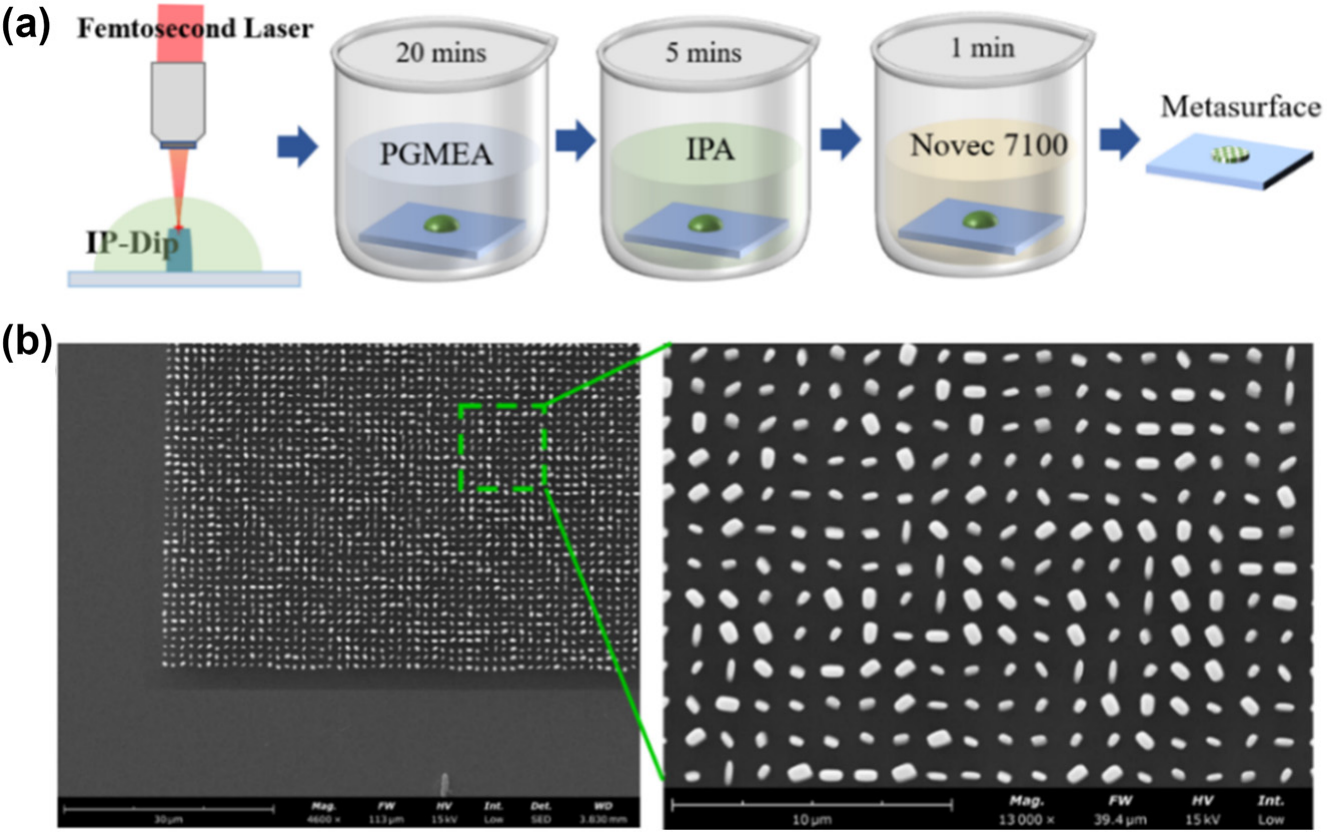
Sample processing flow and the SEM images. (a) The process of processing using 3D TPP printing. (b) SEM images of the sample.

To further experimentally validate the holographic display performance of the proposed metasurface, we designed a metasurface hologram, which consists of 700 × 700 pillars with varying length, width, and rotation angle. The metasurface sample is fabricated by utilizing two-photon polymerization 3D printing technology (Photonic Professional GT2). The processing flow is shown in Figure 5, in order to accurately control the length and width of the processed pillars, we first tested and analyzed the relationship between different processing parameters and the geometric size of the pillars. By adjusting the laser energy, scan speed, and hatching distance and precompensating the design size, we obtained accurate processing parameters that can achieve the four target structure sizes. After the two-photon polymerization is completed, the sample is immersed in the PGMEA developer solution for 20 min and the IPA solution for 5 min. These two steps are to remove the unpolymerized photoresist and clean the developer solution, respectively. Finally, in order to avoid the collapse of intercolumn agglomerates due to surface tension when IPA evaporates, the sample is then soaked in engineered fluid (methoxy-nonafluorobutane, Novec 7100) for 1 min and then taken out to dry. Because Novec 7100 has very low surface tension, it is able to maintain the mechanical properties of the column. The SEM image of the fabricated sample is shown in Figure 5(b). The image on the right is a magnified view of the metasurface, in which the four sizes of meta-atoms used can be clearly distinguished. Each meta-atom maintained a good aspect ratio and did not collapse.

**Figure 6:**
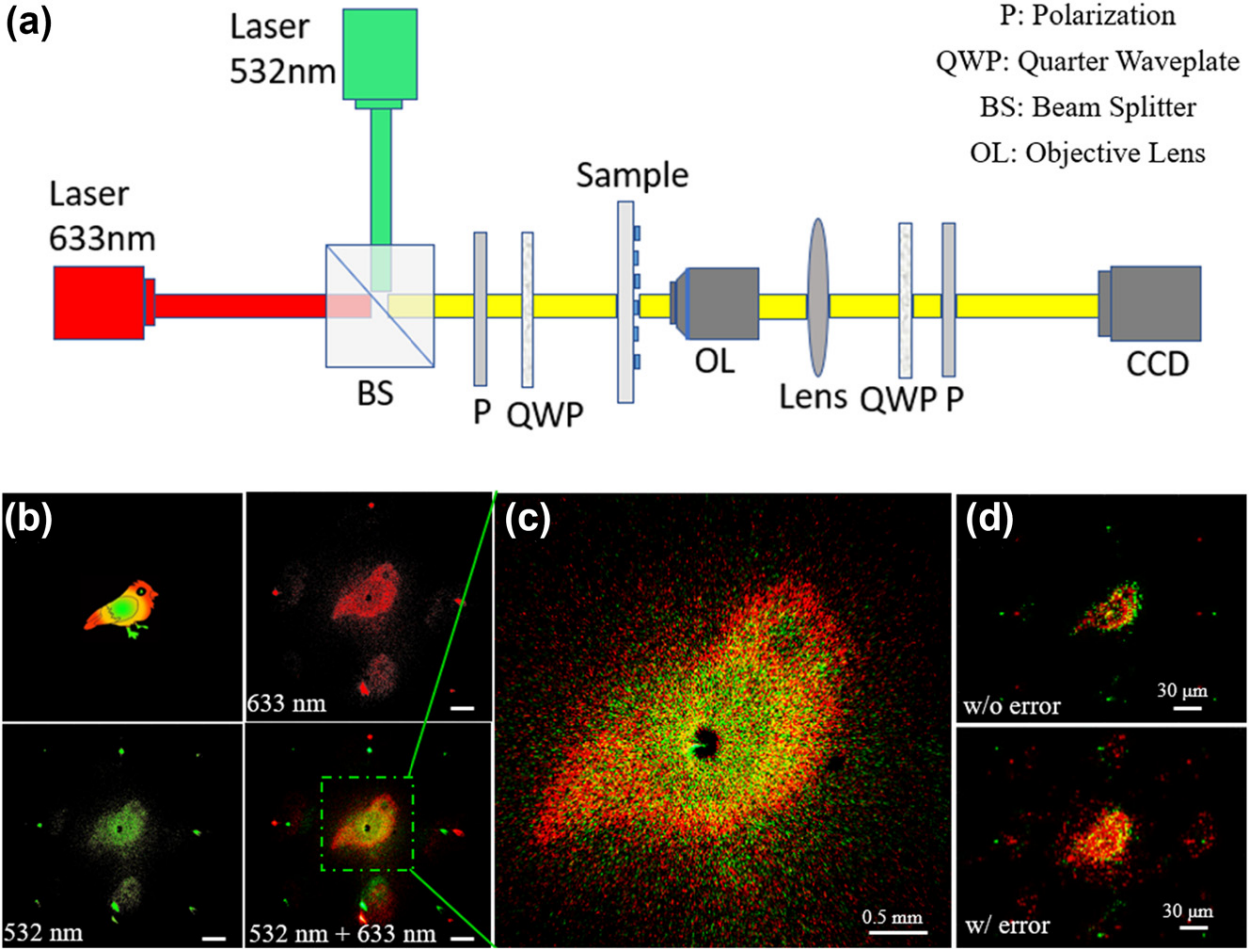
Experimental results. (a) Experimental setup for color meta-holograms. (b) Target image and experimental reconstructed images. Scale bars: 1 mm. (c) Magnified image. (d) Simulation reconstructed images without and with introducing fabrication errors.

The experimental setup is exhibited in Figure 6(a). The incident light beams emitted from two semiconductor lasers with wavelengths of 532 nm and 633 nm are combined by a beam-splitting prism and convert to circularly polarized light (CP) by a broadband linear polarizer (LP) and a quarter-wave plate (QWP). The diameter of combined laser beam spot is greater than 2 mm, which is larger than the size of the metasurface, so it can be directly illuminated onto the metasurface without beam expander. The near-field light after metasurface is collected by a 40 × microscope objective, forming a holographic image behind the back focal plane. Then a glass with a black spot on it is placed at this position to block the focused background light. Subsequently, the image is imaged onto a camera through an achromatic lens, where the QWP and LP are used to extract the cross-circularly polarized light.

The captured reconstructed images are shown in Figure 6(b-b). A colored bird is used as a target image (the upper left image of Figure 6(b)), which is separated into two red and green images for phase calculation and encoding. Reconstructed images are taken under separate and simultaneous illumination with laser beams of two wavelengths. All these reconstruction results show that the higher-order diffraction is visible due to the large period of metasurface. However, the energy is primarily concentrated in the first order, making the reconstructed images of higher orders being less obvious. Figure 6(c) is a magnification of the color reconstructed image of the first order in Figure 6(b). All parts of the bird’s body in the reconstructed image can be reconstructed relatively clearly with less color crosstalk, which is almost consistent with the target image in Figure 6(b). The strong noise may come from the errors introduced during fabrication, such as material shrinkage during the photo-curing process, improper setting of printing parameters, etc., which may lead to deviations in the structural size, shape, or surface roughness of the metasurface. This ultimately leads to the poor quality of the reconstructed image and even a decrease in the polarization conversion efficiency. To confirm this, we performed simulation analysis using a metasurface of 50×50 pixels. Without introducing size errors to the length and width of meta-atoms and introducing random ±10 nm errors, the reconstructed images are shown in Figure 6(d). It can be seen that the size error causes the phase pairs corresponding to the two wavelengths to deviate from the design value, which will lead to more serious noise.

In our experiment, the diffraction efficiency is defined as the power of the reconstructed image divided by the power of the incident light. The diffraction efficiencies measured at wavelengths 532 nm and 633 nm were 11 % and 14 %, respectively. Theoretically, the diffraction efficiency can be further improved, such as using nanoparticle-embedded resin [28] to increase the refractive index to reduce the requirement for aspect ratio and then process meta-atoms with higher PCE, or by reducing the period of meta-atom to eliminate high-order diffraction. The quality of the reconstructed images can also be further improved. For example, by using more phase levels to reconstruct detailed information, but this would require more adequate and complete response data from meta-atoms, which could be achieved by using meta-atoms with higher degrees of freedom, or by using more pixels, but this will require more processing time.

In our proof-of-concept work, considering the complexity of meta-atom structures used, only two wavelengths were multiplexed to achieve multicolor holography. In fact, our method can be extended to more wavelengths. For example, for a three-wavelength holographic display, *m* ^3^ phase pairs are required. By using our method, it can be reduced to *m* ^2^ phase pairs. In this case, we only need to find a richer meta-atom library to realize these *m* ^2^ phase pairs and then combine it with the PB phase theory to achieve three-wavelength multiplexing holographic display.

## 4 Conclusions

In summary, 3D TPP technology is used to fabricate color metasurface holograms. We proposed a design principle for dual-wavelength multiplexing color metasurface holograms based on the combination of propagation phase and geometric phase. Without spatial multiplexing, the high-resolution characteristics of the metasurface hologram can be retained. By introducing the rotation angle, the requirements for the number of meta-atoms can be reduced, which reduces the design difficulty of multifunctional metasurface devices. By optimizing the laser power, scanning speed, and hatching distance in the TPP-based 3D printing technology, the manufacturing of meta-atoms with a minimum line width of 250 nm and an aspect ratio of 16:1 was achieved. The experimental reconstruction of the sample shows that the designed metasurface hologram can display bright and clear color holographic images under the simultaneous irradiation of lasers with wavelengths of 532 nm and 633 nm, which is very consistent with our expectations. Our work not only demonstrates the feasibility of 3D TPP printing technology in preparing metasurface samples in the visible light band but also the proposed metasurface design method provides potential applications in the fields of holographic display, optical encryption, anticounterfeiting, etc. Furthermore, through 3D TPP technology, more complex meta-atoms of different heights or continuous free shapes [29] can be processed to realize multifunctional metasurface devices such as high-performance achromatic lenses [30], [31], chiral metasurfaces [32], [33], BIC metasurfaces [34], [35], and large-capacity metasurface holograms [36], [37], thereby promoting the application of metasurface technology.
